# A three-dimensional investigation of mandibular deviation in patients with mandibular prognathism

**DOI:** 10.1186/s40902-023-00372-y

**Published:** 2023-01-20

**Authors:** Kazuaki Osawa, Jun Nihara, Hideyoshi Nishiyama, Kojiro Takahashi, Ayako Honda, Chihiro Atarashi, Ritsuo Takagi, Tadaharu Kobayashi, Isao Saito

**Affiliations:** 1 Division of Orthodontics, Faculty of Dentistry & Graduate School of Medical and Dental Sciences, Niigata University, 2-5274 Gakkocho-dori, Chuo-Ku, Niigata, 951-8514 Japan; 2 Division of Oral and Maxillofacial Radiology, Faculty of Dentistry & Graduate School of Medical and Dental Sciences, Niigata University, Niigata, Japan; 3 Division of Maxillofacial Surgery, Faculty of Dentistry & Graduate School of Medical and Dental Sciences, Niigata University, Niigata, Japan; 4 Division of Reconstructive Surgery, Faculty of Dentistry & Graduate School of Medical and Dental Sciences, Niigata University, Niigata, Japan

**Keywords:** Jaw deviation, Cluster analysis, Mandibular prognathism, 3-Dimensional analysis, Skull base and menton

## Abstract

**Background:**

Craniofacial disharmony in cases of jaw deformity associated with abnormal lateral deviation of the jaw mostly involves both the maxilla and mandible. However, it has been still difficult to capture the jaw deviation aspect in a 3-dimensional and quantitative techniques. In this study, we focused on 3-dimensional mandibular morphology and position of the condylar head in relation to the base of the skull in patients with mandibular prognathism, one of the most common jaw deformities. We used cluster analysis to quantify and classify deviation and clarified its characteristics. We also investigated the degree of correlation between those findings and menton (Me) deviation measured on frontal cephalograms, which is a conventional indicator of jaw deformity.

**Results:**

Findings obtained from 100 patients (35 men, 65 women) were classified into the following three groups based on mandibular morphology and condylar position relative to the skull base. Then, reclassification using these parameters enabled classification of cluster analysis findings into seven groups based on abnormal jaw deviation characteristics. Comparison among these seven groups showed that the classification criteria were ramus height, mandibular body length, distance from the gonion to the apex of the coronoid process, and the lateral and vertical positions of the mandible. Weak correlation was also found between Me deviation on frontal cephalograms and each of the above parameters measured on 3D images.

**Conclusions:**

Focusing on mandibular morphology and condylar position relative to the skull base in patients with mandibular prognathism, we used cluster analysis to quantify and classify jaw deviation. The present results showed that the 3D characteristics of the mandible based on mandibular morphology and condylar position relative to the skull base can be classified into seven groups. Further, we clarified that Me deviation on frontal cephalograms, which has been used to date, is inadequate for capturing jaw deviation characteristics.

## Background

The number of patients who have chief compliant of an improvement in skeletal disharmony is increasing recently [1]. A nationwide survey in Japan revealed that 67.6% of orthognathic patients was mandibular prognathism [2]. Those patients have frequently showed horizontal skeletal deviations in addition to anteroposterior and/or vertical abnormality [3]. Malocclusion or facial asymmetry in patients with jaw deviation is difficult to improve by orthodontic treatment alone, so that surgical orthodontic treatment is indicated in such cases [4].

Jaw deviation usually involves both the maxilla and mandible. To date, however, various types of jaw deviation have been relatively challenging to visualize using three-dimensional (3D) techniques [5]. It is reported that two-dimensional cephalometric prediction such as STO (Surgical Treatment Objectives) [6] is still popular but not proper to precisely analyze remarkable facial symmetric cases. Thus, treatment planning for patients with jaw deviation remains challenging. In addition, previous study stated that asymmetric bony tissue was often hidden by soft tissue and might be difficult to detect from facial appearance in patients with mandibular prognathism and jaw deviation [7]. Therefore, it is often challenging to determine the optimal configuration of skeletal tissue to ensure soft tissue symmetry in line with the treatment needs.

Jaw deviation is primarily understood as disharmony in the lateral direction on a frontal view. This phenomenon is particularly conspicuous in the chin region, so deviation of the menton (Me) on frontal cephalograms has been used as an indicator to quantify jaw deviation [8]. However, many jaw deformities accompanied by jaw deviation result in morphological asymmetry of both the chin region and the posterior mandibular ramus (i.e., the gonion). Frontal cephalograms, two-dimensional (2D) analytic method, are not considered to be highly reliable since their images may be enlarged or distorted, affected by the position of the head during imaging, and there may be overlap of skeletal structures [9]. From this point of view, 3D analysis is therefore essential for capturing the detailed characteristics of jaw deviation [10]. A few reports have attempted to quantify jaw deviation using 3D computed tomography (CT) images [9, 11, 12], but none of them has classified the characteristics of jaw deviation.

Cluster analysis is an effective method to analyze patients who have a range of different morphologies, as it involves classification into a number of groups based on objective numerical criteria and then analyzing the characteristics of the resulting groups. This qualitative research technique is the best statistical method even for dividing patients with jaw deviation, who exhibits a high degree of variation in craniofacial morphology, into groups based on their its characteristics [13]. On the other hand, jaw deviation is a form of maxillo-mandibular disharmony and involves a complex combination of various factors that affect not only the skeletal tissue, but also the soft tissue. So, the characteristics of jaw deviation might not be captured accurately if the same method of evaluation is used for this wide variety of structures. We also considered that these skeletal and soft tissue features of maxillary and mandibular components should be separated each other for measurements to clarify the characteristics of jaw deviation related with facial asymmetry. For this reason, the maxilla, mandible, and soft tissues have been classified as different regions. Especially the mandibular deviation has been reported to play a crucial role in facial asymmetry and show a wide variety of states [10]. Accordingly, to quantify the features of jaw deviation and identify similar patterns would contribute to standardization of treatment protocols for managing these cases.

In this study, we focused on mandibular morphology and the position of the condylar head in relation to the base of the skull (hereinafter, condylar position relative to the skull base) in patients with mandibular prognathism. We then used cluster analysis to attempt to quantify and classify the features of deviation, divided the patients into groups based on the similarity of their 3D mandibular morphology, and then determined the characteristics of mandibular morphology and condylar position relative to the skull base in these groups. Additionally, we investigated the correlation of Me deviation on 2D frontal cephalograms with mandibular morphology and condylar position relative to the skull base on 3D images.

## Methods

### Subjects

Subjects comprised 100 patients (35 men, 65 women; mean age 22 years 5 months ± 8 years 3 months) diagnosed with mandibular prognathism at the Department of Orthodontics at Niigata University Medical and Dental Hospital from 2009 to 2019. Patients with congenital abnormalities (e.g., cleft lip and palate), syndromes that affected craniofacial morphology, or a history of trauma were excluded from the study.

3D CT images and frontal cephalograms were obtained for all patients during the clinical examination. CT imaging was performed using a multi-detector-row CT scanner (Aquilion, Toshiba, Tokyo, Aquilion ONE, Toshiba, Tokyo, Ingenuity CT, Philips, Netherlands). Patients were placed in the supine position with the mouth closed, positioned parallel to Reid’s base line, and imaging was performed with a tube voltage of 120 kV and a tube current of 54–150 mA. The scanning range was from the superior margin of the orbit to below the mental region (i.e., including the Me) with 0.5– and 1.0–mm slice thickness and 0.3– and 0.5–mm interslice interval. The CT images were converted into DICOM format, then imported to a personal computer and used to create multiplanar reconstruction (MPR) images. The MPR images were created and morphological measurements were performed using the 3D morphological measurement software ZioCube (Ziosoft, Tokyo).

And for morphological analysis, frontal cephalograms were taken under condition that the focal point was set to a distance of 1.5 m from an ear rod. The patient was seated with the Frankfurt horizontal (FH) plane as parallel as possible to the floor. Then, ear rods were placed in the left and right external acoustic meatus, after which the position of the head was fixed. The patient was instructed to achieve the maximal intercuspal position and ensure that their upper and lower lips were touching lightly, and then imaging was performed.

### Three-dimensional morphological measurements

**Fig. 1:**
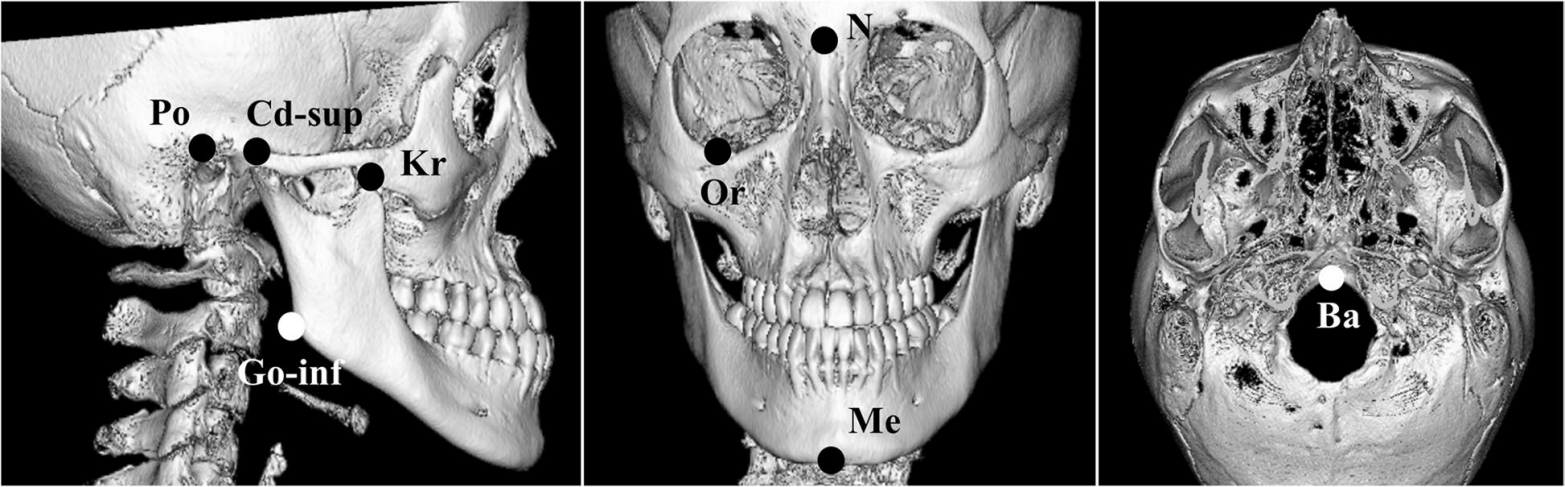
Measurement points. Po: Most superior point of the external acoustic meatus; Cd-sup: Most superior point of the condylar head; Go-inf: Most inferior point on the mandibular angle; Kr: Apex of the coronoid process; N: Most anterior point on the frontonasal suture; Or: Most inferior point on the inferior orbital margin; Me: Most inferior point on the mandibular symphysis; Ba: Most inferior point above the anterior margin of the foramen magnum

First, we created a coordinate system for performing morphological measurements. The xyz coordinate system was established by first defining a horizontal reference plane (xy plane) as the plane passing through the bilateral orbitales (Or, Or′) and the midpoint between the bilateral porions (Po, Po′). The mid-sagittal reference plane (yz plane) was defined as the plane perpendicular to the xy plane and passing through the most inferior point above the anterior margin of the foramen magnum (Ba) and the most anterior point on the frontonasal suture (N). Lastly, the coronal reference plane (xz plane) was defined as the plane perpendicular to both the xy and yz planes that passes through Ba. This coordinate system was used to measure the following 13 points using the method described by Nagai et al. [14] based on the cephalometric landmarks shown in Fig. 1.

- N: Most anterior point on the frontonasal suture
- Ba: Most inferior point above the anterior margin of the foramen magnum
- Po, Po′: Most superior point of the external acoustic meatus
- Or, Or′: Most inferior point on the inferior orbital margin
- Me: Most inferior point on the mandibular symphysis
- Cd-sup, Cd-sup′: Most superior point of the condylar head
- Kr, Kr′: Apex of the coronoid process
- Go-inf, Go-inf′: Most inferior point on the mandibular angle

Next, the xyz coordinate values for each of the measured points were subjected to affine transformation, after which 3D coordinates were determined and superimposed onto the xyz reference coordinate system, and the following reference items were calculated.

a (a′) Ramus height: Distance from Cd-sup (Cd-sup′) to Go-inf (Go-inf′)
b (b′) Body length: Distance from Go-inf (Go-inf′) to Me
c (c′) Cd-Me (mandibular length): Distance from Cd-sup (Cd-sup′) to Me
d (d′) Coronoid (from the gonion to the apex of the coronoid process): Distance from Kr (Kr′) to Go-inf (Go-inf′)
e (e′) Cd-MSP (distance from the mid-sagittal reference plane to the condylar head): Distance from the mid-sagittal reference plane to Cd-sup (Cd-sup′)
f (f′) Cd-CP (distance from the coronal reference plane to the condylar head): Distance from the coronal reference plane to Cd-sup (Cd-sup′)
g (g′) Cd-FH (distance from the horizontal reference plane to the condylar head): Distance from the horizontal reference plane to Cd-sup (Cd-sup′)

Furthermore, the differences in the absolute values of the abovementioned measurements between the left and the right were calculated to capture the degree of asymmetry and were then used to set the following seven analysis items.

1. Ramus height-diff: |a-a′|
2. Body length-diff: |b-b′|
3. Cd-Me-diff: |c–c′|
4. Coronoid-diff: |d-d′|
5. Cd-MSP-diff: |e-e′|
6. Cd-CP-diff: |f-f′|
7. Cd-FH-diff: |g-g′|

The ramus height-diff, body length-diff, Cd-Me-diff, and coronoid-diff analysis items indicate the asymmetry of mandibular morphology, while Cd-MSP-diff, Cd-CP-diff, and Cd-FH-diff indicate asymmetry of the condylar position relative to the skull base.

Only one author (K.O.) set the coordinate system and performed all measurements, and this method of analysis was tested for measurement errors before research data were collected. Specifically, 3D CT images from 10 cases measured by the same individual were randomly selected, an interval of 1 week or more was allowed to elapse, the coordinate system was set again and the measurements were repeated. The measurement error was calculated using Dahlberg’s formula and yielded extremely low values of 0.05 to 0.23.

Based on the measured values for each of the seven analysis items obtained for each patient, we performed cluster analysis using the Ward method in the statistical analysis software program R (ver. 3.3.2, R Foundation for Statistical Computing, Vienna) and created dendrograms. We used the dendrograms to divide the patients into groups, and statistically analyzed the mean values of the analysis items in each group using the Steel–Dwass test to elucidate the characteristics of each group. The level of significance was set to 5%, and statistical analysis was performed using the statistical analysis software JMP (ver. 11.0, SAS Institute Japan K.K., Tokyo). Based on the results obtained from the cluster analyses performed considering mandibular morphology and condylar position relative to the skull base, we reclassified the patients so as to combine the two different perspectives for each patient. We then performed statistical analysis of the mean values of the analysis items in each group using the Steel–Dwass test to elucidate the characteristics of each reclassified group.

**Fig. 2:**
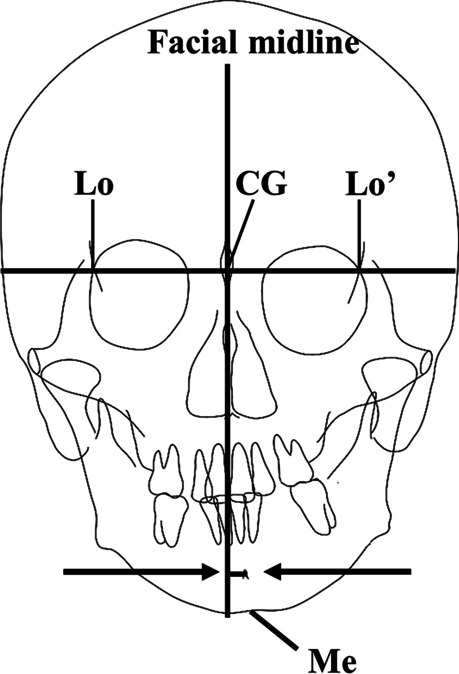
Measurement of Me deviation (perpendicular distance from the facial midline to Me)

Among the groups in which jaw deviation was observed, we used Spearman’s rank correlation coefficients to investigate the correlation of Me deviation on frontal cephalograms, which is frequently used as a conventional indicator of jaw deviation, with mandibular morphology and condylar position relative to the skull base obtained from the analysis in this study. To measure Me deviation, we set the facial midline on a frontal cephalogram. First, we determined the points of intersection of the left and right lateral orbital margins (Lo-Lo′) and the oblique line, then drew a line connecting the two. The line perpendicular to this connecting line that passed through the crista galli of the ethmoid bone was defined as the facial midline. We then measured the distance between the facial midline and Me (Fig. 2).

## Results

### Quantitative classification based on cluster analysis

**Fig. 3:**
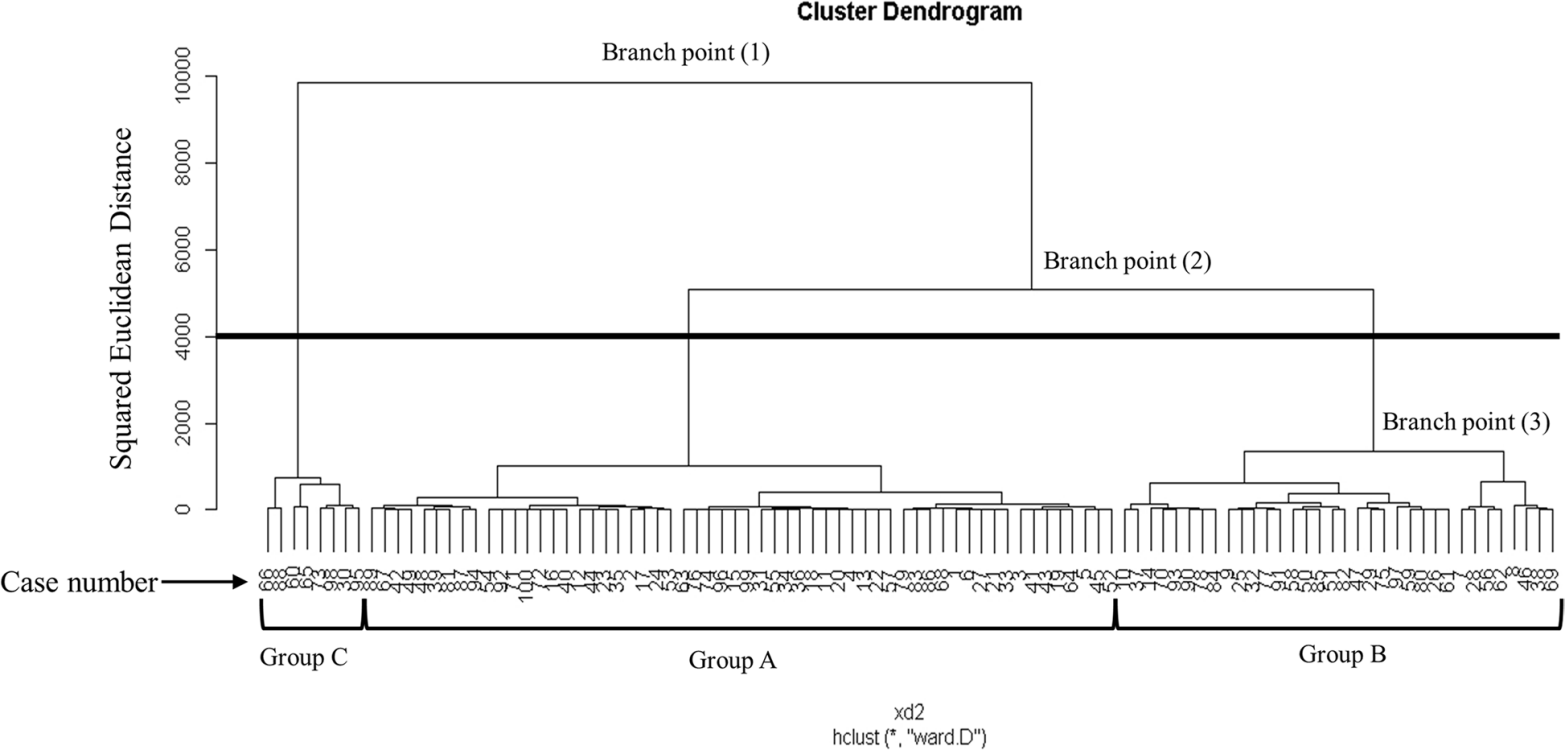
Dendrogram from cluster analysis based on mandibular morphology. Branch point (1): squared Euclidean distance 10000. Branch point (2): squared Euclidean distance 5000. Branch point: squared Euclidean distance 1000. Solid horizontal line indicates the cutoff part

The dendrogram in Fig. 3 shows the cluster analysis results for the four mandibular morphology items (i.e., ramus height-diff, body length-diff, Cd-Me-diff, and coronoid-diff). The number of groups obtained, the squared Euclidean distance between each of the branch points for each group, the sample size bias in each group, and the similarity between the groups were all examined, and the graph was transected between branch points (2) and (3), as shown in Fig. 3, giving a total of three groups: group A included 58 patients; group B, 34; and group C,8.

**Fig. 4:**
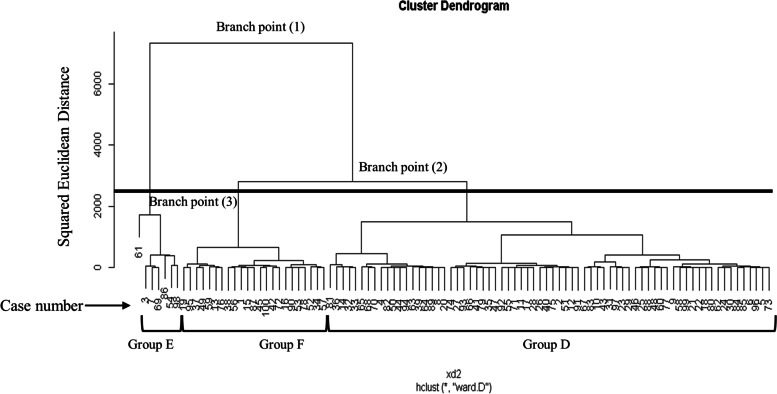
Dendrogram from cluster analysis based on condylar position relative to the skull base position. Branch point (1): squared Euclidean distance 7500. Branch point (2): squared Euclidean distance 2500. Branch point (3) squared Euclidean distance 1700. Solid horizontal line indicates the cutoff part

The dendrogram in Fig. 4 shows the cluster analysis results for the items of condylar position relative to the skull base (i.e., Cd-MSP-diff, Cd-CP-diff, and Cd-FH-diff). The number of groups obtained, the squared Euclidean distance between each of the branch points for each group, the sample size bias in each group, and the similarity between the groups were all examined, and the graph was transected between branch points (2) and (3), as shown in Fig. 4, giving a total of three groups: group D included 70 patients; group E, 7; and group F, 23.

### Comparison of mean values and standard deviations for the analysis items within and between groups

The multigroup comparison results for the three cluster analysis groups for the mandibular morphology analysis items are shown in Table 1.

1. Ramus height-diff: This difference was statistically significantly smaller in group A (1.38 ± 1.06 mm) than in the other two groups, and significantly smaller in group B (3.11 ± 2.03 mm) than in group C (7.41 ± 2.74 mm).
2. Body length-diff: This difference was significantly smaller in group A (1.03 ± 0.86 mm) than in group B (3.20 ± 1.79 mm), but there was no significant difference in the results between group C (3.03 ± 2.38 mm) and group A or B.
3. Cd-Me-diff: This difference was significantly smaller in group A (1.83 ± 1.20 mm) than in group B (4.32 ± 1.84 mm) and group C (7.41 ± 3.68 mm), but there was no significant difference in the results between groups B and C.
4. Coronoid-diff: This difference was significantly larger in group C (4.14 ± 2.68 mm) than in groups A (1.58 ± 1.06 mm) and B (2.00 ± 1.58 mm), but there was no significant difference in the results between groups A and B.

The multigroup comparison results for the three cluster analysis groups for the analysis items of condylar position relative to the skull base are shown in Table 2.

(5) Cd-MSP-diff: This difference was significantly larger in group E (3.69 ± 2.52 mm) than in groups D (1.40 ± 1.07 mm) and F (1.01 ± 0.72 mm), but there was no significant difference in the results between groups D and F.
(6) Cd-CP-diff: There were no significant differences observed between groups D (2.13 ± 1.47 mm), E (6.12 ± 3.72 mm), and F (2.73 ± 1.71 mm).
(7) Cd-FH-diff: This difference was significantly smaller in group D (0.86 ± 0.58 mm) than in groups E (3.05 ± 1.73 mm) and F (3.02 ± 0.65 mm), but there was no significant difference in the results between groups E and F.

**Table 1:** Intergroup comparisons of analysis items based on mandibular morphology

Analysis item	Group A		Group B		Group C		Group A vs Group B	Group A vs Group C	Group B vs Group C
	Mean	SD	Mean	SD	Mean	SD			
Ramus height-diff	1.38	1.06	3.11	2.03	7.41	2.74	***	***	**
Body length-diff	1.03	0.86	3.20	1.79	3.03	2.38	***	n.s	n.s
Cd-Me-diff	1.83	1.20	4.32	1.84	7.41	3.68	***	**	n.s
Coronoid-diff	1.58	1.06	2.00	1.58	4.14	2.68	n.s	**	*

^*^p < 0.05; **p < 0.01; ***p < 0.001; *n.s*. Not significant

**Table 2:** Intergroup comparisons of analysis items based on condylar position relative to the skull base

Analysis item	Group D		Group E		Group F		Group D vs Group E	Group D vs Group F	Group E vs Group F
	Mean	SD	Mean	SD	Mean	SD			
Cd-MSP-diff	1.40	1.07	3.69	2.52	1.01	0.72	*	n.s	**
Cd-CP-diff	2.13	1.47	6.12	3.72	2.73	1.71	n.s	n.s	n.s
Cd-FH-diff	0.86	0.58	3.05	1.73	3.02	0.65	**	***	n.s

^*^p < 0.05; **p < 0.01; ***p < 0.001; *n.s*. Not significant

### Quantitative classification based on mandibular morphology and condylar position relative to the skull base

Patients were classified according to mandibular morphology and condylar position relative to the skull base. Based on the results obtained from the above cluster analysis, we reclassified the subjects so as to combine these two different perspectives for each patient. The results are shown in Table 3. Only 1 patient each belonged to both groups C and E and groups C and F, so they were excluded from analysis. We were therefore able to classify 98 subjects into seven groups numbered 1 to 7.

**Table 3:** Reclassification results based on characteristics of mandibular morphology and condylar position relative to the skull base

		Mandibular morphology		
		Group A	Group B	Group C
Condylar position vs skull base	Group D	Group 1 (39)	Group 4 (25)	Group 7 (6)
	Group E	Group 2 (3)	Group 5 (3)	1
	Group F	Group 3 (16)	Group 6 (6)	1

The breakdown for each of the groups shows that the greatest number of patients (39) were classified into group 1, indicating that they exhibited no deviation of mandibular morphology or condylar position relative to the skull base; in other words, these patients had mandibular prognathism without asymmetry. By comparison, 59 patients (classified into groups 2 to 7) exhibited deviations of either mandibular morphology or condylar position relative to the skull base, meaning that these patients exhibited asymmetry. Among the groups in which asymmetry was observed, the greatest number of patients (25) belonged to group 4, followed by group 3 with 16, and groups 6 and 7 with 6 each. Among the groups with asymmetry, the fewest patients were in groups 2 and 5 (3 patients each).

A comparison of the mean values and standard deviations for the analysis items within each group is shown in Table 4, and an intergroup comparison is shown in Table 5.

1. Ramus height-diff: This difference was significantly larger in groups 4 (2.95 ± 1.75 mm) and 7 (7.07 ± 3.05 mm) than in group 1 (1.21 ± 0.83 mm). Groups 4 and 7 had the largest difference in mandibular ramus height between the left and the right.
2. Body length-diff: This difference was significantly larger in group 4 (3.09 ± 1.56 mm) than in groups 1 (1.02 ± 0.89 mm) and 3 (1.04 ± 0.83 mm). Group 4 had the largest difference in mandibular body length between the left and the right.
3. Cd-Me-diff: This difference was significantly larger in groups 4 (4.48 ± 1.91 mm) and 6 (4.53 ± 1.10 mm) than in groups 1 (1.90 ± 1.19 mm) and 3 (1.62 ± 1.27 mm). Group 1 and group 3 had the smallest differences in mandibular length between the left and the right.
4. Coronoid-diff: This difference was significantly larger in groups 7 (5.04 ± 2.50 mm) than in groups 1 (1.61 ± 1.12 mm), 3 (1.55 ± 0.88 mm), and 4 (1.86 ± 1.49 mm). Group 7 had the largest difference in distance from the gonion to the apex of the coronoid process between the left and the right.
5. Cd-MSP-diff: No significant differences was observed between any of the groups.
6. Cd-CP-diff: No significant differences was observed between any of the groups.
7. Cd-FH-diff: This difference in group 3 (2.98 ± 0.68 mm) was significantly larger than in groups 1 (0.82 ± 0.63 mm), 4 (0.80 ± 0.51 mm), and 7 (1.31 ± 0.30 mm), while the difference in group 6 (2.93 ± 0.37 mm) was significantly larger than in groups 1 (0.82 ± 0.63 mm) and 4 (0.80 ± 0.51 mm). Group 3 and group 6 had the largest difference in vertical position of the condylar head between the left and the right.

**Table 4:** Mean values and standard deviation of analysis items in each group

Analysis item	Group 1		Group 2		Group 3		Group 4		Group 5		Group 6		Group 7	
	Mean	SD	Mean	SD	Mean	SD	Mean	SD	Mean	SD	Mean	SD	Mean	SD
Ramus height-diff	1.21	0.83	0.96	0.40	1.89	1.40	2.95	1.75	2.18	1.59	4.22	2.76	7.07	3.05
Body length-diff	1.02	0.89	1.08	0.58	1.04	0.83	3.09	1.56	4.56	1.62	2.99	2.40	3.57	2.41
Cd-Me-diff	1.90	1.19	2.16	0.78	1.62	1.27	4.48	1.91	2.62	1.59	4.53	1.10	6.67	3.92
Coronoid-diff	1.61	1.12	1.32	1.03	1.55	0.88	1.86	1.49	3.03	1.90	2.09	1.58	5.04	2.50
Cd-MSP-diff	1.47	1.04	4.05	0.56	1.16	0.75	1.30	1.14	3.78	3.71	0.73	0.44	1.37	0.89
Cd-CP-diff	1.96	1.48	5.41	3.58	2.37	1.60	2.31	1.47	8.80	0.20	3.48	1.79	2.49	1.24
Cd-FH-diff	0.82	0.63	3.94	0.82	2.98	0.68	0.80	0.51	1.29	0.49	2.93	0.37	1.31	0.30
Me deviation	2.27	1.72	2.33	1.03	2.97	1.92	5.14	2.21	4.17	1.03	5.33	4.96	8.67	3.98

**Table 5:** Comparison of analysis items among groups 1–7

Analysis item	Subject	*P* value	Significance
Ramus height-diff	Group 1 vs Group 4	0.0019	**
	Group 1 vs Group 7	0.0078	**
Body length-diff	Group 1 vs Group 4	< .0001	***
	Group 3 vs Group 4	0.0008	***
Cd-Me-diff	Group 1 vs Group 4	< .0001	***
	Group 1 vs Group 6	0.0111	*
	Group 3 vs Group 4	0.0024	**
	Group 3 vs Group 6	0.0287	*
Coronoid-diff	Group 1 vs Group 7	0.0124	*
	Group 3 vs Group 7	0.014	*
	Group 4 vs Group 7	0.0399	*
Cd-MSP-diff	n.s		n.s
Cd-CP-diff	n.s		n.s
Cd-FH-diff	Group 1 vs Group 3	< .0001	***
	Group 1 vs Group 6	0.0019	**
	Group 3 vs Group 4	< .0001	***
	Group 3 vs Group 7	0.0084	**
	Group 4 vs Group 6	0.0037	**

^*^p < 0.05; **p < 0.01; ***p < 0.001; *n.s*. Not significant

**Fig. 5:**
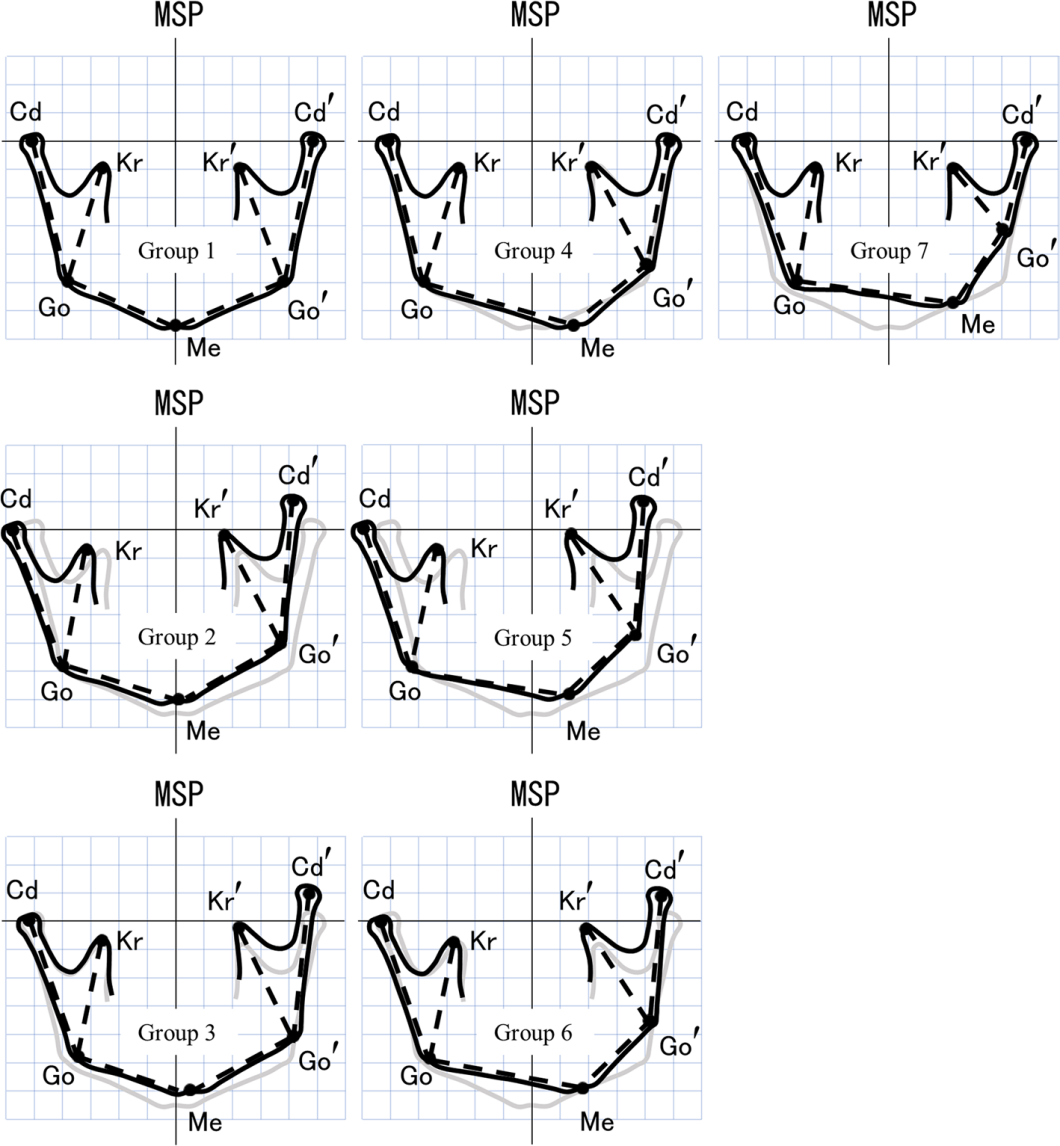
Conceptual diagram representing the characteristics of each group. (Gray line shows the diagram of Group1 as a symmetrical shape of mandible.)

Based on the abovementioned statistical analysis results, we were able to describe the characteristics of the seven groups classified in this study and create conceptual diagrams to represent these characteristics (Fig. 5).

- Group 1: Mandibular morphology and condylar position relative to the skull base are approximately symmetrical
- Group 2: Mandibular morphology is approximately symmetrical and there is both lateral and vertical deviation of the condylar position relative to the skull base
- Group 3: Mandibular morphology is approximately symmetrical and there is vertical deviation of the condylar position relative to the skull base
- Group 4: There are large differences in ramus height and body length between the left and right, but condylar position relative to the skull base is approximately symmetrical
- Group 5: There is a large difference in ramus height and body length between the left and right, and there is lateral and vertical deviation of condylar position relative to the skull base
- Group 6: There is a large difference in ramus height and body length between the left and right, and there is vertical deviation of the condylar position relative to the skull base
- Group 7: There is a large difference in ramus height and distance from the gonion to the apex of the coronoid process between the left and right, but condylar position relative to the skull base is approximately symmetrical

### Correlation between Me deviation and each of the analysis items in the jaw deviation groups

We investigated the correlation between Me deviation and each analysis item in groups 2 to 7, in which subjects exhibited asymmetry of either mandibular morphology or condylar position relative to the skull base (Table 6).

**Table 6:** Results of correlation between Me deviation and each analysis items

		Spearman’s rank correlation coefficient	*P* value	Significance
Me deviation vs	Ramus height-diff	0.45	0.0003	***
	Body length-diff	0.27	0.038	*
	Cd-Me-diff	0.66	< .0001	***
	Coronoid-diff	-0.09	0.5195	n.s
	Cd-MSP-diff	-0.01	0.9219	n.s
	Cd-CP-diff	0.09	0.5098	n.s
	Cd-FH-diff	-0.28	0.0324	*

^*^p < 0.05; **p < 0.01; ***p < 0.001; *n.s.* Not significant

A significant correlation was observed between Me deviation and ramus height-diff, body length-diff, Cd-Me-diff, and Cd-FH-diff, but no correlation was observed between Me deviation and coronoid-diff, Cd-MSP-diff, or Cd-CP-diff. Among the items for which a significant correlation was observed, a positive correlation was observed for ramus height-diff, body length-diff, and Cd-Me-diff, while a negative correlation was observed for Cd-FH-diff. The correlation coefficient showed moderate correlation (0.66) with Cd-Me-diff, but weak correlations with ramus height-diff (0.45), Cd-FH-diff (-0.28), and body length-diff (0.27).

## Discussion

### Patients and methods

The present study on mandibular deviation focused on only patients with mandibular prognathism. They were not classified using Me deviation on frontal cephalograms, though previous studies have tried to use to quantify jaw deviation [10]. The mandibular deviation has been observed as an asymmetry of not only Me but also the gonial regions [15], so that we attempted to quantify jaw deviation in patients with mandibular prognathism from the two perspectives of mandibular morphology and condylar position relative to the skull base. As a result, our study would enable depiction of features of jaw deviation that have not been considered to date and greatly contribute to the quantification of jaw deviation.

Setting the mid-sagittal reference plane is extremely important when performing 3D evaluation of jaw deviation and it is essential to set anatomical reference points that are as unaffected by jaw deviation as possible in order to ensure appropriate evaluation [16]. Thiesen et al. noted that the mid-sagittal reference plane should a clinically feasible and highly reproducible reference line [12]. Taking this into account, we looked for reference points that were as unaffected by jaw deviation as possible, easy to establish, and highly reproducible. In this study, for the mid-sagittal reference plane, we selected the plane perpendicular to the FH plane and passing through both N and Ba. N and Ba are both located on the midline and are some distance from the measurement points, so they are the least affected by jaw deviation. We also considered the possibility that FH may be affected by jaw deviation. However, FH is frequently used in clinical situation. For example, during evaluation of the face, we usually utilize images in which the FH plane is parallel to the floor. Therefore, we decided to adopt it as one of the reference planes in this study. Although the anterior nasal spine (ANS) was used as a reference point in previous report [17], considering possible deviation of the maxilla also occur, we used no maxillary reference points when setting any of the reference planes and instead opted for measurement points on stable structures close to the cerebral cranium.

In the present study, only linear measurements were used and the differences between measurement values on the left and the right were analyzed in order to evaluate deviation properties. The reason for this is that the classification of characteristics would become more complicated if both linear and angular measurements were included in the measurement items, which would make it more difficult to capture the features of the deviation.

### Jaw deviation and cluster analysis

Numerous studies to date have focused on the composition of deviation [9–11, 18, 19], although the deviation features are complicated and there is enormous variation, so this issue has not yet been adequately clarified. In the present study, we focused on mandibular morphology and condylar position relative to the skull base. We then used cluster analysis to simplify the complicated features and attempted to quantify and classify jaw deviation. Cluster analysis [13] is a method that facilitates division of various types of data into populations based on objective numerical standards, so we believe it is suited to the classification of complex jaw deviation. A few previous studies have classified the characteristics of maxillofacial morphology using cluster analysis. Hwang et al. used frontal cephalograms and performed cluster analysis to classify 100 patients diagnosed with facial asymmetry into five groups that exhibited definite characteristics [18]. Meanwhile, Baek et al. used 3D CT images to perform cluster analysis of 43 patients diagnosed with facial asymmetry and reported that they could be classified into four groups [11]. However, one of the four groups obtained by Baek et al. included only two individuals, suggesting that the classification may not have been statistically reliable. For this reason, in the present study, we set a sample size of 100 patients in order to avoid any issues in achieving accurate classification due to a small sample size after cluster analysis.

The branch points on the dendrogram obtained by cluster analysis were based on the squared Euclidean distance. The closer the branch points were on the dendrogram, the greater the similarity between arbitrary clusters, while greater distances between branch points indicated lower similarity. In other words, the shorter the long axis of the dendrogram, the higher the intercluster similarity becomes. As a result, when selecting the transection site on the dendrogram to divide subjects into groups, it is advisable to ensure that the squared Euclidean distance is adequately long, and that the number of clusters ensures that each cluster has distinct characteristics. When we created the dendrogram for the mandibular morphology items in this study, a squared Euclidean distance of approximately 10,000 points (branch (1) in Fig. 3) was first divided into two clusters. We then selected a squared Euclidean distance of approximately 5000 points (branch (2) in Fig. 3) in one of these clusters, and then divided it in two again. Meanwhile, when we created the dendrogram for items of condylar position relative to the skull base in this study, a squared Euclidean distance of approximately 7500 points (branch (1) in Fig. 4) was first divided into two clusters. We then selected a squared Euclidean distance of approximately 2500 points (branch (2) in Fig. 4) in one of these clusters, and then divided it in two again. If the dendrogram were transected at a point inferior to branches (3) on Figs. 3 and 4, the squared Euclidean distance would decrease, and the similarity between each cluster would increase, which would make the characteristics more indistinct. For this reason, by transecting the dendrogram between branches (2) and (3) on Figs. 3 and 4, we obtained three clusters each and determined that we had obtained a suitable number of clusters for clinical application.

### Classification of jaw deviation

To divide the patients into groups based on the quantification of jaw deviation and deviation characteristics, we first investigated mandibles in which structural asymmetry tends to arise. Since jaw deviation commonly affects both the maxilla and mandible in cases of jaw deformity associated with facial asymmetry, we originally believed that we should focus on both structures during the investigation. However, we presumed that this would complicate the quantification, and that the morphological characteristics would not be expressed accurately. For this reason, in the present study, we focused on only the mandible in cases of mandibular prognathism to simplify the characteristics to be captured, and we attempted to classify the condition from the two perspectives of the mandibular morphology and condylar position relative to the skull base.

For mandibular morphology, we used the Steel–Dwass test to clarify the characteristics of each group obtained by cluster analysis. We found that all mandibular morphology items (ramus height-diff, body length-diff, Cd-Me-diff and coronoid-diff) were significantly smaller in group A. Ramus height-diff, body length-diff and Cd-Me-diff were significantly larger in group B, and there was a moderate difference in ramus height and a large difference in body length between the left and right sides. Ramus height-diff, Cd-Me-diff and coronoid-diff were significantly larger in Group C, and it was clear that this group included cases with large differences between the left and right sides in terms of ramus height and distance from the gonion to the apex of the coronoid process. Meanwhile, in terms of condylar position relative to the skull base, we found that the Cd-MSP-diff and Cd-FH-diff were significantly smaller in group D, and this group included cases in which virtually no differences in condylar head position were observed between the left and right sides. In group E, Cd-MSP-diff and Cd-FH-diff were significantly larger, and there was lateral and vertical deviation of the condylar head in these cases. However, in group F, Cd-FH-diff was significantly larger, and this group included cases with vertical deviation of the condylar head.

Based on the various cluster analysis results for mandibular morphology and condylar position relative to the skull base, we reclassified individual cases in order to integrate these two perspectives. These results showed that the mandibular morphology was approximately symmetrical and the condylar head was not deviated in relation to the base of the skull group 1, whereas there was deviation of mandibular morphology or condylar position relative to the skull base, or both in groups 2 to 7. Group 1 included 39 patients, which was approximately 40% of the total, while the remaining 60% of cases exhibited some kind of asymmetry or deviation. Severt et al. reported that 34% of patients with jaw deformity exhibit clinically obvious facial asymmetry [20], while Oguri et al. performed an analysis in which they defined lateral deviation as ≥ 4 mm of deviation in the maxillary and mandibular midline and reported that lateral deviation was observed in 48.6% of patients in whom surgical orthodontic treatment was indicated [1]. Compared with these studies, our study had a higher percentage of patients with asymmetry, although we believe that enabled detection of cases in which jaw deviation was previously difficult to diagnose, and particularly cases with deviation of the condylar head.

These cases could be classified into six groups (groups 2 to 7) in which there was asymmetry or deviation of the mandibular morphology or condylar position relative to the skull base, or both. Among these groups, groups 2 and 3 had deviation of only the condylar head and the deviation was both lateral and vertical in group 2 but only vertical in group 3. The frequencies of occurrence for these groups were 3% and 16%, respectively. We focused on only the mandible in this study, but groups 2 and 3 were characterized by a highly symmetric mandible although it was positioned asymmetrically on the left and right sides. We therefore believe that there may have been concomitant horizontal cant of occlusal plane or maxillary deviation. In addition, in group 4, the position of the condylar head was approximately symmetrical, but the mandibular morphology, and particularly the difference in mandibular body length between the left and the right, was large, appearing with a frequency of 25%. This was the highest frequency among the groups with asymmetry. In this group, we believe that the asymmetry arose in the mandibular morphology only, and therefore, a type of ramus osteotomy is likely indicated in these cases. However, Nishida et al. reported that the indications for ramus osteotomy are limited and that there may be residual postoperative deviation in cases with extreme deviation [21], so correcting mandibular morphology by means of bimaxillary surgery, genioplasty, or mandibular angleplasty should be investigated. Accordingly, considering the degree of improvement in not only skeletal asymmetry but also soft tissue asymmetry among the groups obtained during this study, we believe that ramus osteotomy and bimaxillary surgery should be investigated in these groups.

For groups 5 and 6, the respective frequencies of occurrence were not high at 3% and 6% respectively. However, there were large differences in both ramus height and body length between the left and right sides. In addition, group 5 had both lateral and vertical displacement of the condylar head, whereas group 6 had only vertical displacement. As we used absolute values for differences in mandibular morphology and condylar head position between the left and right sides in this study, we cannot determine the direction of deviations. However, if the directions of mandibular morphological asymmetry and deviation of condylar position relative to the skull base were the same, then the jaw deviation would likely be severe. If the mandibular morphology were asymmetrical and the direction of the condylar position relative to the skull base were retrograde, aspects of so-called reverse cant cases would likely be present [22] and many of these cases would meet the indications for maxillo-mandibular surgery.

In group 7, the position of the condylar head was approximately symmetrical, but there were large differences in mandibular body length and coronoid process length between the left and right sides. The frequency of occurrence for this group was 6%. Coronoid hyperplasia and coronoid hypoplasia are examples of conditions with a large difference between the left and right coronoid processes, but both are relatively rare disorders. According to Galie et al. [23], unilateral coronoid hyperplasia may be common in patients with facial asymmetry. Yoshida et al. [24] noted that first and second branchial arch syndrome and Treacher-Collins syndrome are examples of congenital disorders that cause coronoid hypoplasia, while acquired disorders include systemic scleroderma, trauma, tumors, and chronic inflammation. None of the patients included in the present study had obvious congenital abnormalities or syndromes that would affect craniofacial morphology. However, there was a significantly greater difference in the values for ramus height-diff and coronoid-diff mandibular morphology in group 7 than in the other groups. Therefore, these findings indicate that, despite the absence of congenital abnormalities, there were cases of jaw deformity with large differences between the left and right sides in the vertical morphology of the ramus and coronoid process.

Studies to date have investigated the mandibular morphology and condylar position relative to the skull base separately [25–27], but none has attempted to classify jaw deviation using both of these characteristics in combination. Obwegeser et al. reported classifying mandibular asymmetry due to unilateral mandibular hyperplasia into three types: unilateral hyperplasia of the condylar head, condylar neck, and mandibular ramus; unilateral elongation of the mandibular body; and a mixture of these two types [25]. We believe that this classification resembles the groups with a large difference in ramus height and the groups with a difference in body length between the left and right sides. Obwegeser et al. classified mandibular asymmetry characteristics from 2D images and histopathology images, whereas our morphological classification is based on distance measurements on 3D images. Jaw deviation causes 3D morphological abnormalities in maxillofacial bony tissue, so we expect that the classification derived in this study will be useful for capturing jaw deviation.

In terms of asymmetry of the condylar position relative to the skull base, Yorozuya et al. reported that the horizontal distance between the mid-sagittal reference plane and condylar head was shorter on the deviated side [26]. In our study, we detected asymmetry using differences between the left and right sides, and performed classification based on the measurement results. Thus, we did not classify our findings into those on a deviated side or non-deviated side and perfect comparison is not possible. However, our study also revealed a group in which the condylar head is displaced laterally, so we believe that the jaw deviation characteristics indicated by Yorozuya et al. may correspond to cases included in this group. Meanwhile, using 2-dimentional axial cephalometric projection, O’Byrn found that the condylar head was in a posterosuperior position on the deviated side, meaning that there was a difference between the condylar position relative to the skull base on left and the right sides in the anteroposterior and vertical directions [27]. In our present study, we found no significant differences in anteroposterior condylar position relative to the skull base on the left and right sides, so this was not expressed as a group characteristic, although it was consistent with the finding that there were differences in the vertical direction between the left and right sides. In the present study, we grouped patients based on 3D characteristics of jaw deviation from the two perspectives of the morphological characteristics of the mandible itself and the positioning of the condylar head in the maxillofacial region as an index. This approach should contribute to an accurate understanding of jaw deviation in cases of jaw deformity associated with facial asymmetry.

In this work, the three groups each that were obtained by cluster analysis of mandibular morphology and condylar position relative to the skull base were reclassified into seven groups. Then, we performed a statistical analysis comparing the groups to clarify the characteristics of each one. Results showed that although there were groups in which it was possible to elucidate characteristics for which they were statistically significant differences, there were also some groups between which no statistically significant differences were observed. Specifically, group 2 was characterized by asymmetry of condylar position relative to the skull base, groups 5 and 6 were characterized by asymmetry of the mandibular morphology and condylar position relative to the skull base in both directions, and group 7 was characterized by asymmetrical mandibular morphology. No statistically significant differences were detected during the comparison between these groups. After reclassifying the groups, there were less than 10 patients in several of the groups and we believe that it is highly likely that the small patient populations affected the statistical analysis. To avoid having groups with small sample sizes during the study, we decided to include 100 patients, but ultimately there were multiple groups that consisted of less than 10% of the overall sample size. Although we cannot rule out the possibility that the number of cases was inadequate, the results showing numerous groups with a small number of cases appear to reflect the high degree of variation in jaw deviation. We expect a greater number of cases will be needed to more accurately clarify the characteristics of each group in further study.

### Correlation between Me deviation and each analysis item

We used groups 2 to 7, in which asymmetry of the mandibular morphology or condylar position relative to the skull base was observed, to investigate the correlation between each analysis item and Me deviation on frontal cephalograms. The results showed that the strongest correlation was observed between Me deviation and Cd-Me-diff, but the correlation was moderate with a correlation coefficient of 0.66. In addition, even for the measurement items with significant correlations, the correlation coefficients were low at 0.3 to 0.4, so the characteristics of jaw deviation could be captured by Me deviation in only those cases characterized by differences in total mandibular length. This in turn shows that Me deviation can capture only a fraction of the features of mandibular jaw deviation. Frontal cephalograms are a standardized imaging modality and can be used for comparisons between individuals or evaluation of chronological changes in an individual, so they are frequently used as a means to effectively capture longitudinal data on the morphology of the craniofacial and maxillofacial regions. However, as frontal cephalograms are a 2D evaluation, one of the disadvantages is that the image may be affected by the position of the head during imaging. Accordingly, we believe that frontal cephalograms play a useful role in screening for the presence of jaw deviation in jaw deformities, but 3D evaluation by means of 3D CT imaging is ultimately essential for evaluating complex jaw morphology, such as the position of structures, rotation, and distortion in cases of facial asymmetry with multiple aspects, as well as for reaching an accurate diagnosis and devising a treatment plan [28, 29].

## Conclusion

In this study, we focused on mandibular morphology and condylar position relative to the skull base in 100 patients with mandibular prognathism. We used cluster analysis to attempt to quantify and classify jaw deviation. This resulted in the following seven group classifications.

- Group 1: Mandibular morphology and condylar position relative to the skull base are approximately symmetrical.
- Group 2: Mandibular morphology is approximately symmetrical and there is lateral and vertical deviation of condylar position relative to the skull base.
- Group 3: Mandibular morphology is approximately symmetrical, and there is vertical deviation of condylar position relative to the skull base.
- Group 4: There is a moderate difference in ramus height and a large difference in body length between the left and right sides, but condylar position relative to the skull base is approximately symmetrical.
- Group 5: There is a moderate difference in ramus height and a large difference in body length between the left and right sides, and there is lateral and vertical deviation of condylar position relative to the skull base.
- Group 6: There is a moderate difference in ramus height and a large difference in body length between the left and right sides, and there is vertical deviation of condylar position relative to the skull base.
- Group 7: There are large differences in ramus height and distance from the gonion to the apex of the coronoid process between the left and right sides, but condylar position relative to the skull base is approximately symmetrical.

The above classification indicated that an effective quantitative analysis of jaw deviation would focus on ramus height, body length, and distance between the gonion and the apex of the coronoid process relevant to mandibular morphology, and on condylar position relative to the skull base as well.

With the exception of a few cases, the horizontal measurement of Me deviation on frontal cephalograms was not well correlated with measurement items pertaining to asymmetrical morphology, indicating that Me deviation is inadequate for analyzing characteristics of jaw deviation.

## Data Availability

The datasets generated during and/or analyzed during the current study are available from the corresponding author on reasonable request.

## Abbreviations

MPR: Multiplanar reconstruction
FH: Frankfurt horizontal
CT: Computed tomography
2D: Two-dimensional
3D: Three-dimensional
Me: Menton

## References

[1] Oguri Y, Naganuma K, Harada F, Watanabe A, Yamaki M, Saito C, Takagi R, Saito I (2010). Clinical survey of surgical-orthodontics cases treated in the past decade at the Orthodontic Department, Niigata University Medical and Dental Hospital. Jpn J Jaw Deform.

[2] Kobayashi T, Saito C, Inoue N, Ohata N, Kawamura H, Goto S, Goto M, Shiratsuchi Y, Susami T, Tanne K, Hashimoto K, Moriyama K, Amagasa T, Himuro T, Tonoki M (2008). Treatment of jaw deformity: a nationwide survey of the situation in Japan. Jpn J Jaw Deform.

[3] Saito I, Watanabe N, Yamaki M (2010). Facial asymmetry and deviated jaw and/or occlusion. Niigata Dent J.

[4] Proffit WR, Fields HW, Sarver DM, Proffit WR, Fields HW, Sarver DM (2013). Combined surgical and orthodontic treatment. Contemporary orthodontics.

[5] Wakamatsu T, Yamaki M, Hanada K, Hayashi T, Saito I (2007). Three-dimensional evaluation of morphological asymmetry of maxillofacial complex in patients showing mandibular prognathism with facial asymmetry. Jpn J Jaw Deform.

[6] Wolford L, Hilliard FW, Dugan DJ, Wolford L, Hilliard FW, Dugan DJ (1985). The initial STO. Surgical treatment objective.

[7] Chen YJ, Yao CC, Chang ZC, Lai HH, Yeh KJ, Kok SH (2019). Characterization of facial asymmetry in skeletal Class III malocclusion and its implications for treatment. Int J Oral Maxillofac Surg.

[8] Haraguchi S, Takada K, Yasuda Y (2002). Facial asymmetry in subjects with skeletal Class III deformity. Angle Orthod.

[9] Yanez-Vico RM, Iglesias-Linares A, Torres-Lagares D, Gutierrez-Perez JL, Solano-Reina E (2011). Three-dimensional evaluation of craniofacial asymmetry: an analysis using computed tomography. Clin Oral Investig.

[10] You K-H, Lee K-J, Lee S-H, Baik H-S (2010). Three-dimensional computed tomography analysis of mandibular morphology in patients with facial asymmetry and mandibular prognathism. Am J Orthod Dentofacial Orthop.

[11] Baek C, Paeng J-Y, Lee JS, Hong J (2012). Morphologic evaluation and classification of facial asymmetry using 3-dimensional computed tomography. J Oral Maxillofac Surg.

[12] Thiesen G, Mota Freitas MP, Araujo EA, Gribel BF, Kim KB (2018). Three-dimensional evaluation of craniofacial characteristics related to mandibular asymmetries in skeletal Class I patients. Am J Orthod Dentofacial Orthop.

[13] Honda A, Takahashi K, Nihara J, Takagi R, Kobayashi T, Saito I (2018). Classification of frontal facial patterns in patients with mandibular prognathism. Niigata Dent J.

[14] Nagai Y, Nishiyama H, Nihara J, Tanaka R, Yamaki M, Hayashi T, Saito I (2013). A study on reproducibility of three-dimensional measurement for an evaluation of craniofacial morphology. J Jpn Soc Bone Morphom.

[15] Kiji N, Matsui S, Kiji Y, Katayama K, Otsuka Y, Kiyomura H (1997). Effects of mandibular asymmetry on soft tissue outline. Jpn J Jaw Deform.

[16] Zhang D, Wang S, Li J, Zhou Y (2018). Novel method of constructing a stable reference frame for 3-dimensional cephalometric analysis. Am J Orthod Dentofacial Orthop.

[17] Shin SM, Kim Y-M, Kim N-R, Choi Y-S, Park S-B, Kim Y-I (2016). Statistical shape analysis-based determination of optimal midsagittal reference plane for evaluation of facial asymmetry. Am J Orthod Dentofacial Orthop.

[18] Hwang H-S, Youn I-S, Lee K-H, Lim H-J (2007). Classification of facial asymmetry by cluster analysis. Am J Orthod Dentofacial Orthop.

[19] Kim J-Y, Jung H-D, Jung Y-S, Hwang C-J, Park H-S (2014). A simple classification of facial asymmetry by TML system. J Craniomaxillofac Surg.

[20] Severt TR, Proffit WR (1997). The prevalence of facial asymmetry in the dentofacial deformities population at the University of North Carolina. Int J Adult Orthod Orthognath Surg.

[21] Nishida E, Tamura T, Tonogi M, Ohki H, Shimizu N (2013). Evaluation of postoperative frontal face in facial asymmetry cases following sagittal splitting ramus osteotomy. Jpn J Jaw Deform.

[22] Uesugi S, Yonemitsu I, Kokai S, Omura S, Ono T (2014). Morphological feature of subjects with facial asymmetry with the frontal occlusal plane inclined toward the contralateral side of the mandibular deviation. Jpn J Jaw Deform.

[23] Galie M, Consorti G, Tieghi R, Denes SA, Fainardi E, Schmid JL, Neuschl M, Clauser L (2010). Early surgical treatment in unilateral coronoid hyperplasia and facial asymmetry. J Craniofac Surg.

[24] Yoshida K, Mori S, Yokoi T, Kuroiwa Y, Kurita K (2015). A case of unilateral hypoplasia of the coronoid process during long-term follow-up. Jpn J Oral Maxillofac Surg.

[25] Obwegeser HL, Makek MS (1986). Hemimandibular hyperplasia–hemimandibular elongation. J Maxillofac Surg.

[26] Shibazaki-Yorozuya R, Yamada A, Nagata S, Ueda K, Miller AJ, Maki K (2014). Three-dimensional longitudinal changes in craniofacial growth in untreated hemifacial microsomia patients with cone-beam computed tomography. Am J Orthod Dentofacial Orthop.

[27] O'Byrn BL, Sadowsky C, Schneider B, BeGole EA (1995). An evaluation of mandibular asymmetry in adults with unilateral posterior crossbite. Am J Orthod Dentofac Orthop.

[28] An S, Lee J-Y, Chung CJ, Kim K-H (2017). Comparison of different midsagittal plane configurations for evaluating craniofacial asymmetry by expert preference. Am J Orthod Dentofacial Orthop.

[29] Katsumata A, Fujishita M, Maeda M, Ariji Y, Ariji E, Langlais R (2005). 3D-CT evaluation of facial asymmetry. Oral Surg Oral Med Oral Pathol Oral Radiol Endod.

